# Beneficial Effects of *Tinospora cordifolia* on Blood Profiles in Male Mice Exposed to Lead

**DOI:** 10.4103/0971-6580.68341

**Published:** 2010

**Authors:** V. Sharma, D. Pandey

**Affiliations:** Bioscience and Biotechnology Department, Banasthali University, Banasthali - 304 022, Tonk, Rajasthan, India

**Keywords:** Hematology, lead nitrate, mice, *Tinospora cordifolia*

## Abstract

This study was carried out to evaluate *in vivo* protective role of aqueous extract of stem and leaves of *Tinospora cordifolia* (TC) on the toxic effects of lead on the hematological values. The lead-treated (5 mg/kg body weight, intraperitonially, once daily) male albino mice concurrently received either *T. cordifolia* stem or leaves extracts (400 mg/kg body weight, orally, once daily) for the duration of 30 days. The animals exposed to lead showed significant decrease in RBC and Hb level. Significant decline in WBC, DLC, and PCV was also noticed. Increase in MCV values displaying reciprocal relationship with RBC, PCV, and Hb values in lead-treated mice were also recorded. These influences of lead were prevented by concurrent daily administration of *T. cordifolia* stem and leaves extract. These results suggested that simultaneous supplementation of *T. cordifolia* protects against lead intoxication.

## INTRODUCTION

*Tinospora cordifolia* (family Menispermacea; commonly known as Guduchi or Giloy), a glabrous climbing shrub, is widely used in folk and ayurvedic system of medicine in India since ancient times.[1,2] The whole plant is used for therapeutic purpose. It is reported that the plant is bitter but nontoxic and also has ability to scavenge free radicals. A variety of constituents used for drug preparation have been isolated from the plant. They belong to different classes such as alkaloids, diterpenoids lactones, glycosides, steroids, sesquiterpenoides, phenolics, aliphatic compounds, and polysaccharides. The alkaloids tinosporin, tinosporic acid, and tinosporol rich in protein, calcium, and phosphorus have been identified in leaves.[3,4] Its remarkable and notable medicinal properties such as antidiabetic, antiperiodic, antispasmodic, antimalarial, antiflammatory, antiarthritic, antioxidant, antiallergic, antistress, antileprotic, hepatoprotective, immunomodulatory, blood purification, and antineoplastic activities are well documented.[2,4,7]

Despite the fact that *T. cordifolia* is an important medicinal plant, so far to our knowledge, no attempt was made to study its regulatory role in lead toxicity, in particular. Lead is the most common environment pollutant and has been shown to present health problems following exposure. Until now the studies regarding regulation of lead toxicity are restricted to some chelating agents[8,9] and few antioxidants such as vitamin C and vitamin E.[10,11] However, most of the conventional metal chelating agents and antioxidants have been reported to possess toxic side-effects or disadvantages.[12] Thus, there has been increased interest in the therapeutic potential of plant products or medicinal plants having beneficial role in reducing lead poisoning. Keeping, this view in mind, the present study was planned to determine the effects of aqueous extract of stem and leaves of *T. cordifolia* on lead-induced hematological alternation following lead exposure.

## MATERIALS AND METHODS

### Chemicals

Lead nitrate Pb(NO_3_)_2_ and all other chemicals used in this study were of analytical grade and were purchased from reliable firms like SRL and BDH chemical (India), MERCK (Germany).

### Preparation of crude extract of *T. cordifolia*

The *T. cordifolia* plant was collected from the medicinal garden of Banasthali University, Rajasthan, India, and identified by a plant taxonomist of the institute. The plant materials (stem and leaves) were thoroughly washed with distilled water, shade dried and cut into small pieces, and powdered separately using laboratory homogenizer. Known quantities of these powdered materials were extracted separately using distilled water as a solvent. The extracts were then filtered through filter paper and concentrated on water bath. Finally after complete evaporation of the solvent, the residue were weighed and stored at 4°C and used to treat the animals as needed.

### Animals

Male Swiss albino mice weighing approximately 15–30 g were obtained from Haryana agricultural university, Hissar, India. The animals were acclimatized for 7 days prior to experiment. The institutional ethics committee approved the experimental protocols. All the animals used in this study were placed in stainless steel cages in an air conditioned room maintained at temperature of 25±2 ° C and 12 h light and dark schedule. Throughout the experiment, the animals were provided standard food pellet (Hindustan lever ltd.) and water *ad libitum*. Essential cleanness conditions were also maintained.

### Experimental design

Mice were divided into six groups of six each and the groups were as follows:

- Group I - Control animals (no treatment)
- Group II – Lead nitrate (5mg/kg body weight)
- Group III – TC stem extract (400 mg/kg body weight)
- Group IV – TC leaves extract (400 mg/kg body weight)
- Group V – TC stem extract (400 mg/kg body weight) + lead nitrate
- Group VI – TC leaves extract (400 mg/kg body weight) + lead nitrate

All the above groups except group I were treated once daily for the period of 30 days. After the administration of the last dose, the animals were given rest overnight and then on the next day, they were sacrificed under light ether anesthesia. The blood was collected by cardiac puncture with anticoagulant for hematological parameters. The various blood profiles viz., WBC and RBC,[13] Hb,[14] PCV,[15] and DLC (Leishmania staining method) were analyzed. Lead concentrations in blood was estimated by an atomic absorption spectrophotometer.

### Statistical analysis

Data are expressed as the mean±S.E. Statistical analysis was done using analysis of variance (ANOVA). The level of significance was set at*P* < 0.05.

## RESULTS

The hematological status of animals, i.e., levels of white blood cells (WBC), red blood cells (RBC), hemoglobin (Hb), packed cell volume (PCV), mean corpuscular volume (MCV), mean corpuscular hemoglobin (MCH), mean corpuscular hemoglobin content (MCHC), and differential leucocytes count (DLC) of different groups were assessed and are given in Tables 1 and 2. Lead exposure led to significant rise (*P* < 0.05) in blood lead concentration (2.14±0.17 μg/100 ml), and fall in WBC (3.00±0.58 × 10 ^3^ /mm), RBC (3.00±0.58 × 10 ^6^ /mm), Hb (7.00±0.59 g/dl), and PCV (15.00±0.96%) as compared to respective control values (*P* < 0.05). Increase in MCV (50.97±4.49 cu microns) and decrease in MCH (23.64±2.02 pg) and MCHC (46.75±2.86 g/dl) values were also observed in lead-treated mice. *T. cordifolia* stem and leaf extracts significantly increased (*P* < 0.05) Hb and PCV levels. WBC level increased moderately but not significantly with *T. cordifolia* stem extract. Marginal increase in WBC with TC leaf extract was also observed. No effect of *T. cordifolia* extracts in normal animals on RBC number and blood lead concentration was noted.

**Table 1:** Protective effects of *Tinospora cordifolia* on some hematologial profiles in lead-exposed mice

Groups	WBC	RBC	Hb	PCV	MCV	MCH	MCHC	Blood lead
Control (untreated animals)	4.80±1.06	5.01±0.2	10.60±0.33	19.00±0.21	47.28±2.26	24.37±1.32	51.59±1.31	0.05±0.00
Pb	3.00±0.58^a^	3.00±0.58^a^	7.00±0.59^a^	15.00±0.96^a^	50.97±4.49^a^	23.64±2.02	46.75±2.86	2.14±0.17^a^
TC (stem)	5.60±0.17	4.50±0.22	14.50±0.38^a^	25.80±1.62^a^	57.96±4.46	32.53±1.54	57.70±2.69	0.17±0.03
TC (leaf)	4.86±0.20	4.30±0.24	12.60±0.60^a^	25.20±1.15^a^	59.50±4.09	30.01±2.77	50.12±1.73	0.21±0.01
TC (stem) + Pb	5.10±0.08^b^	4.40±0.08^b^	12.8±0.39^b^	19.00±0.30^b^	42.77±0.86^b^	29.11±0.89^b^	68.11±2.03^b^	1.14±0.18^b^
TC (leaf) + Pb	4.62±0.12^b^	4.32±0.10^b^	10.80±0.39^b^	18.70±0.24^b^	42.53±1.45^b^	24.99±0.64	52.15±6.68	1.34±0.09^b^

TC,*Tinospora cordifolia*; Pb, lead; WBC, white blood cells (×10^3^/mm); RBC, red blood cells (×10^6^/mm; hemoglobin (g/dl); PCV, packed cell volume (%); MCV, mean cell volume (cu microns); MCH, mean cell hemoglobin (pg); MCHC, mean cell hemoglobin concentration (g/dl); blood lead concentration, μg/100 ml; Values are mean±S.E.; *n* = 6;

a *P*< 0.05 compared to control (untreated) animals;

b *P*< 0.05 compared to lead-exposed animals

**Table 2:** Protective effects of *Tinospora cordifolia* on differential leukocyte count in mice treated with lead

Groups	Lymphocyte	Monocyte	Neutrophil
Control (untreated animals)	42.00±0.77	9.0±0.36	24.0±0.73
Pb	36.0±1.18^a^	4.20±0.16^a^	21.5±0.84
TC (stem)	48.0±1.44^a^	14.0±0.73^a^	30.0±1.29^a^
TC (leaf)	45.0±1.06^a^	10.3±0.33	24.0±0.73
TC (stem) + Pb	42.0±1.06^b^	12.0±0.57^b^	26.0±0.93^b^
TC (leaf) + Pb	45±2.89^b^	8.0±0.46^b^	22.5±0.51^b^

Lymphocyte (%); monocyte (%); neutrophil (%); Values are mean±S.E.; *n* = 6;

a P< 0.05 compared to control (untreated) animals;

b P< 0.05 compared to lead-exposed animals

When *T. cordifolia* stem and leaf extracts were administered along with the lead, it was found that both extracts could normalize the levels of WBC (5.10±0.08; 4.62±0.12 × 10 ^3^ /mm), RBC (4.40±0.08; 4.32±0.10 × 10 ^6^ /mm), Hb (12.80±0.39; 10.80±0.39 g/dl), PCV (19.00±0.30; 18.70±0.24%) values by significantly increasing (*P* < 0.05) all the blood profiles. Significant decrease (*P* < 0.05) in MCV (42.77±0.86; 43.53±1.45 cu microns) with both plant extracts, profound increase in (*P* < 0.05) MCHC with TC stem extract and small increase in MCHC with TC leaf was also recorded. Coadministration of *T. cordifolia* stem extract with lead significantly increased (*P* < 0.05) the MHC (29.11±0.89 pg) level, but marginal effect on MHC level with *T. cordifolia* leaf extract with lead was also noticed as compared to lead-treated animals. Significant decrease in blood lead level was also observed with both extracts of plant when given individually but in combination with lead. It was interesting to note that Hb level elevated above the normal values with both plant extracts plus lead. Further MCH and MCHC values were also reached above the normal values with TC stem extract.

Table 2 indicates significant decrease (*P* < 0.05) in values of monocytes (4.20±0.16) and lymphocytes (36.00±1.18), whereas slight decrease in neutrophil count (21.5±0.84) was also observed in lead-treated animals as compared to untreated animals. Administration of *T. cordifolia* stem extract significantly increased (*P* < 0.05) the level of all leucocytes count and TC leaf extract significantly increased (*P* < 0.05) lymphocyte count, while marginal increase in monocyte and neutrophil count with TC leaf extract were also observed as compared to normal animals. Coadministration of *T. cordifolia* stem and leaf extract during lead nitrate exposure leads to normal or significant rise in almost all the leukocyte count. Interestingly, the monocyte and neutrophil values were higher in TC stem plus lead (12. 0±0.57 and 26.0±0.93) and lymphocyte (45.00±2.89) values were higher with TC leaf extract + lead than the untreated animals, which could be due to a significant effect of the plant products.

## DISCUSSION

Lead is a cumulative poison and unlike acute poison, lead poisoning occurs slowly. One of the targets of the lead toxicity is *hematological system*. The result of the present study shows that lead affects this system by inhibiting the heme and hemoglobin synthesis and induces anemia (decreased in RBC, WBC count, PCV values, and Hb concentration) in mice [Table 1]. It is well known that the presence of lead in the organism decreases the level of iron in the blood and causes the decrease in Hb concentration. Farant and Wigfield[16] reported that inhibition of ALAD, a cytosolic sulfydryl enzyme and ferrochelatase by lead resulted in depressed heme synthesis that ultimately led to anemia. Further, Ratcliff[17], Monterio et al.,[18,19] and Hermes-lima et al.[20–22] explained that ALAD inactivation may lead to the accumulation of delta- aminoleuvulinic acid that can cause overproduction of ROS, which could lead to lead-induced oxidative damage in the cell and result in anemia.

The decrease in PVC value in the blood of lead-treated mice indicated the increased destruction of erythrocytes, i.e., decrease in RBC count. These observations are inconsistent with several reports.[23–27] Decrease in WBCs by lead in the present study is in favor with earlier reports, which reported mild leucopenia in mice treated with lead. Resultant erythrocytopenia, leucopenia, and hemoglobinemia are considered as direct toxic effect of lead on the blood cells and hematopoietic system.

Decrease in DLC count by lead was also observed in the present investigation [Table 2]. Lymphocytopenia and neutropenia produced by lead is an indication of immunosuppression in this study. However administration of both extract of *T. cordifolia* produced moderate to significant effect in almost all the blood profiles. Several reports have suggested that *T. cordifolia* is used in the treatment of multiple disorders; it also enriches the blood.[7,28,29] When the *T. cordifolia* extract along with lead was administered, they decreased the toxic effect of lead in blood as compared to lead-treated animals, thus indicating the protective role of the plant extract in lead toxicity. It is further noticed that the *T. cordifolia* extract significantly prevented the influences of lead on differential leukocyte count.

Earlier studies with known antioxidants such as vitamin E and C showed that they aid in reducing the lead toxicity from blood.[27,30] These workers examined antioxidant effect of N-acetylcysteine and Succimer in red blood cells of lead-exposed rats. The results provided that lead-induced oxidative stress in RBCs were reversed by thiol antioxidants (NAC) as well as by chelating agent (Succimer). The present findings clearly suggested that *T. cordifolia* stem and leaves crude extracts are capable of scavenging lead-induced hematological alternations to some extent.
